# Depression-Related Work Disability: Socioeconomic Inequalities in Onset, Duration and Recurrence

**DOI:** 10.1371/journal.pone.0079855

**Published:** 2013-11-20

**Authors:** Jenni Ervasti, Jussi Vahtera, Jaana Pentti, Tuula Oksanen, Kirsi Ahola, Mika Kivimäki, Marianna Virtanen

**Affiliations:** 1 Development of Work and Organizations, Finnish Institute of Occupational Health, Helsinki, Finland; 2 Department of Public Health, University of Turku and Turku University Hospital, Turku, Finland; 3 Department of Epidemiology and Public Health, University College London, London, United Kingdom; 4 Department of Behavioral Sciences, University of Helsinki, Helsinki, Finland; Iran University of Medical Sciences, Iran (Republic of Islamic)

## Abstract

**Objective:**

Depression is a major cause of disability in working populations and the reduction of socioeconomic inequalities in disability is an important public health challenge. We examined work disability due to depression with four indicators of socioeconomic status.

**Methods:**

A prospective cohort study of 125 355 Finnish public sector employees was linked to national register data on work disability (>9 days) due to depressive disorders (International Classification of Diseases, codes F32–F34) from January 2005 to December 2011. Primary outcomes were the onset of work disability due to depressive disorders and, among those with such disability, return to work after and recurrent episodes of work disability due to depression.

**Results:**

We found a consistent inverse socioeconomic gradient in work disability due to depression. Lower occupational position, lower educational level, smaller residence size, and rented (vs. owner-occupied) residence were all associated with an increased risk of work disability. Return to work was slower for employees with basic education (cumulative odds ratio = 1.21, 95% CI: 1.05–1.39) compared to those with higher education. Recurrent work disability episodes due to depression were less common among upper-grade non-manual workers (the highest occupational group) than among lower-grade non-manual (hazard ratio = 1.16, 95% CI: 1.07–1.25) and manual (hazard ratio = 1.14, 95% CI: 1.02–1.26) workers.

**Conclusions:**

These data from Finnish public sector employees show persistent socioeconomic inequalities in work disability due to depression from 2005 to 2011 in terms of onset, recovery and recurrence.

## Introduction

In addition to decreased quality of life, depression causes substantial work impairment and lost work days [1]–[3]. Major depressive disorders ranked second on years lost due to disability in 2010 and the burden of major depressive disorder, measured with disability adjusted life years, has increased over time [4].

Socioeconomic status (SES) represents individuals’ access to social and economic resources, and education, occupation, and income or wealth, have been used as its indicators [5]. There is robust evidence that depression is more common among individuals with low SES [6]. Lower income [7], [8] and lower occupational position [9] are linked to a higher risk of depression and related work disability. However, few studies have studied the association of SES with work disability due to depression taking into account the different components of disability that is, onset, recovery, and recurrence.

The course of depression may vary according to SES in that a lower rate of return to work (RTW) from disability period is seen among those with low SES. To date, findings on work disability due to depression and its outcomes have been heterogeneous, varying according to country and the SES indicator. Virtanen et al. [9], for example, reported lower SES (as measured with occupational position) to be associated with longer time to RTW in a Finnish sample. In contrast, two Dutch studies found higher SES (as measured with educational level) to predict longer duration until RTW [10] and no association between SES (as measured with neighborhood income) and RTW of employees with mood disorders [11]. A third Dutch study [12] demonstrated no association between educational level or income and RTW after major depressive disorder, and a Danish study [13] found no association between educational level and RTW among employees sick listed due to mental health problems. It is unclear whether the absence of associations in some of these studies [12], [13] was due to insufficient statistical power (sample sizes <300).

The evidence on recurrence of depressive disorder was recently summarized in a systematic review [14]. While low SES was a risk factor for the onset of depression, it seemed not to be related to its recurrence. A more recent study on a patient sample, however, found that indicators of higher SES (higher income, higher education, employment) predicted antidepressant response and remission [15]. A higher occupational position was also associated with lower rates of recurrence of all-cause psychiatric work disability among Finnish public sector employees [9]. Furthermore, lower income predicted higher recurrence of sick leave due to common mental disorders in a Dutch sample [16].

Given the controversy around the associations between SES, work disability due to depression and its course, we set out to examine these associations in a large cohort of employees. Our aim was to determine whether socioeconomic inequalities in work disability due to depression exist in terms of onset, recovery and recurrence.

## Methods

### Study Context, Design and Participants

The Finnish Public Sector study cohort consists of employees working for ten municipalities and six hospital districts in Finland [17]. All men and women employed in these organizations for over six months in any year between 1991 and 2005, and from the full spectrum of socioeconomic groups were eligible (n = 151 901). Due to the nature of public sector jobs in Finland, the largest occupational groups are nurses and teachers. Consequently, most of the study participants were women (76%).

For this study, we selected those cohort members (n = 130 533) who were working-aged (18–65 years) and who were not deceased, on disability pension, or on old-age pension at baseline (1 January 2005). Their records on employment and socioeconomic factors were linked to the work disability and drug prescription registers maintained by the Social Insurance Institution of Finland through the unique personal identification codes that are assigned to all citizens in Finland. Linkage to registers was 100% complete, and there was no sample attrition during the follow-up. We excluded 5 178 employees with missing data on at least one indicator of SES resulting in a final analytic sample of 125 355 participants (96% of the original sample).

### Ethics Statement

The Finnish Public Sector Study was approved by the Ethics Committee of the Helsinki and Uusimaa Hospital District.

### Work Disability due to Depression

Information on dates of all periods of absence from work (temporary and permanent disability) due to depression was derived from national registers kept by the Social Insurance Institution of Finland and the Finnish Centre for Pensions. These include sick leaves of over nine days and disability pension data on temporary, permanent, full-time and part-time disability pensions. The main diagnosis for the disability period assigned by the treating physician was available for all sick leaves and disability pensions, and they were coded according to the International Classification of Diseases [18]. From these data, we examined disability due to a depressive episode (F32), a recurrent depressive disorder (F33), and persistent mood disorders (F34) in 2005–2011. We measured:

1. Cumulative days absent from work due to depression per cumulative person years in the period 2005–2011 and annually;
2. Total days absent from work due to depression in the period 2005–2011;
3. Time to RTW (at least for a day) after the disability episode due to depression categorized as <2 months (1–59 days), 2–4 months (60–119 days), 4–8 months (120–239 days), 8–12 months (240–365 days), and >12 months (>365 days);
4. Recurrent disability episode due to depression among those with at least one work disability period: 1 = a new disability episode after the end of the first disability episode; 0 = no disability episodes due to depression, disability pension with diagnosis other than F32–F34, old-age pension, death, end of follow-up.

### SES Indicators

Occupational position, education, residence size, and residence ownership were used as SES indicators in this study. Occupational position at the beginning of follow-up was derived from the employers’ registers and categorized according to the occupational title classification [19] as follows: upper-grade non-manual workers (e.g. physicians, teachers, professionals), lower-grade non-manual workers (e.g. technicians, registered nurses, kindergarten teachers), and manual workers (e.g. cleaners, maintenance workers, kitchen workers).

Statistics Finland provided information on education that was classified as high (tertiary level: polytechnic, university education), intermediate (upper secondary level), or basic (lower secondary level or less: nine years of comprehensive education), using the Finnish Standard Classification of Education 2011 [20]. From the Population Register Centre [21], we derived data on residence size (<70 m^2^; 70–100 m^2^; >100 m^2^) and on residence ownership (owner or renter). These measures of accommodation were regarded as a proxy for income and wealth, an approach also used in previous studies [22], [23].

### Covariates

The covariates were sex, age, chronic somatic disease, and work disability due to a mental and behavioral disorder (ICD-10 codes F00–F99) in 2004. Age and sex were obtained from the employers records. The presence of chronic physical disease at baseline (no, yes) was ascertained from the national health records: 1) The incidence of diagnosed prevalent hypertension, cardiac failure, ischemic heart disease, diabetes, asthma or other chronic obstructive lung disease, and rheumatoid arthritis was obtained from the Drug Reimbursement Register kept by the Social Insurance Institution of Finland; 2) Information regarding malignant tumors diagnosed during the preceding five years was obtained from the Finnish Cancer Register covering all diagnosed cancer cases in Finland.

### Statistical Analysis

To examine the onset of work disability due to depression we used negative binomial regression models and estimated rate ratios (RR) with their 95% confidence interval (CI) for categories of SES indicators.

For time to RTW, a variable with five categories, we used multinomial logistic regression procedure calculating cumulative odds ratios (COR) and their 95% CI.

We used Cox proportional hazard models with recurrent events to examine the associations between SES indicators and recurrence of work disability due to depression. The results are presented as hazard ratios (HR) and their 95% CI. The follow-up was from the end of the prior disability episode (codes F32–F34) to the beginning of the next disability episode (codes F32–F34), disability pension with a diagnosis code other than F32–F34, old age pension, death, or the end of the study (31 December 2011), whichever came first. We used SAS statistical software, version 9.2 for all analyses.

## Results

### Distribution of SES Indicators

A third of the participants had an upper-grade non-manual job, half (49%) had a lower-grade non-manual job and 20% were manual workers (Table 1). Lower-grade non-manual jobs were more common among women (56%) than among men (26%). The study population was relatively well-educated: 54% had completed higher education, and 10% had completed basic education only. Of the study participants, 61% owned their residence, and 39% rented. Smaller and rented residences were slightly more common among male employees than among females. Days absent from work due to depression ranged from 0 to 2556 during the whole seven-year study period (2005–2011). Women were more often absent from work due to depression than men.

**Table 1:** Characteristics of study population.

Study variable	Men (n = 30123)	Women (n = 95232)	All (n = 125355)
**Demographics and covariates**			
Age, mean (SD)	43.7 (10.4)	43.2 (10.5)	43.3 (10.5)
Chronic somatic disease			
Yes	3924 (13.0)	11475 (12.0)	15399 (13.3)
No	26199 (87.0)	83757 (88.0)	109956 (87.7)
Mental disorder as main diagnosis of work disability in 2004^a^			
Yes	664 (2.2)	3602 (3.8)	4266 (3.4)
No	29459 (97.8)	91630 (96.2)	121089 (96.6)
**Indicators of socioeconomic status**			
Occupational position			
Upper-grade non-manual	11318 (37.6)	27595 (29.0)	38913 (31.0)
Lower-grade non-manual	7848 (26.0)	52981 (55.6)	60829 (48.5)
Manual	10957 (36.4)	14656 (15.4)	25613 (20.5)
Educational level			
Basic	4196 (13.9)	8682 (9.1)	12878 (10.3)
Intermediate	11294 (37.5)	33355 (35.0)	44649 (35.6)
Higher	14633 (48.6)	53195 (55.9)	67828 (54.1)
Residence size (m^2^)			
<70	10508 (34.9)	29873 (31.4)	40381 (32.2)
70–100	10193 (33.8)	34693 (36.4)	44886 (35.8)
>100	9422 (31.3)	30666 (32.2)	40088 (32.0)
Residence ownership			
Owner	17846 (59.2)	58524 (61.5)	76370 (60.9)
Renter	12277 (40.8)	36708 (38.5)	48985 (39.1)
**Work disability due to depression (F32–F34)**			
Cumulative days absent from work due to depression during 2005–2011 per person-year	2.8	3.8	3.6

Note. Values are n (%) unless stated otherwise.

a Based on International Classification of Diseases, 10^th^ revision, codes F00–F99.

The Spearman rank-order correlations between the SES indicators were all statistically significant (p<0.001; data not shown). Higher occupational position was moderately correlated with higher educational level (r = 0.61) while the other correlations of SES indicators were small ranging between r = 0.12 and r = 0.17. Compared to those renting accommodation, participants owning residence had more often a residence of >100 m^2^ (43% vs. 15%); were more likely in an upper-grade non-manual jobs (35% vs. 25%) with higher education completed (60% vs. 46%) (p<0.001, *x* ^2^ tests).

As no statistically significant sex × SES indicator interactions were found (p-values ranged between 0.59 and 0.85) in work disability due to depression, further analyses were carried out for men and women combined.

### Socioeconomic Differences in Work Disability due to Depression in 2005–2011

**Figure 1:**
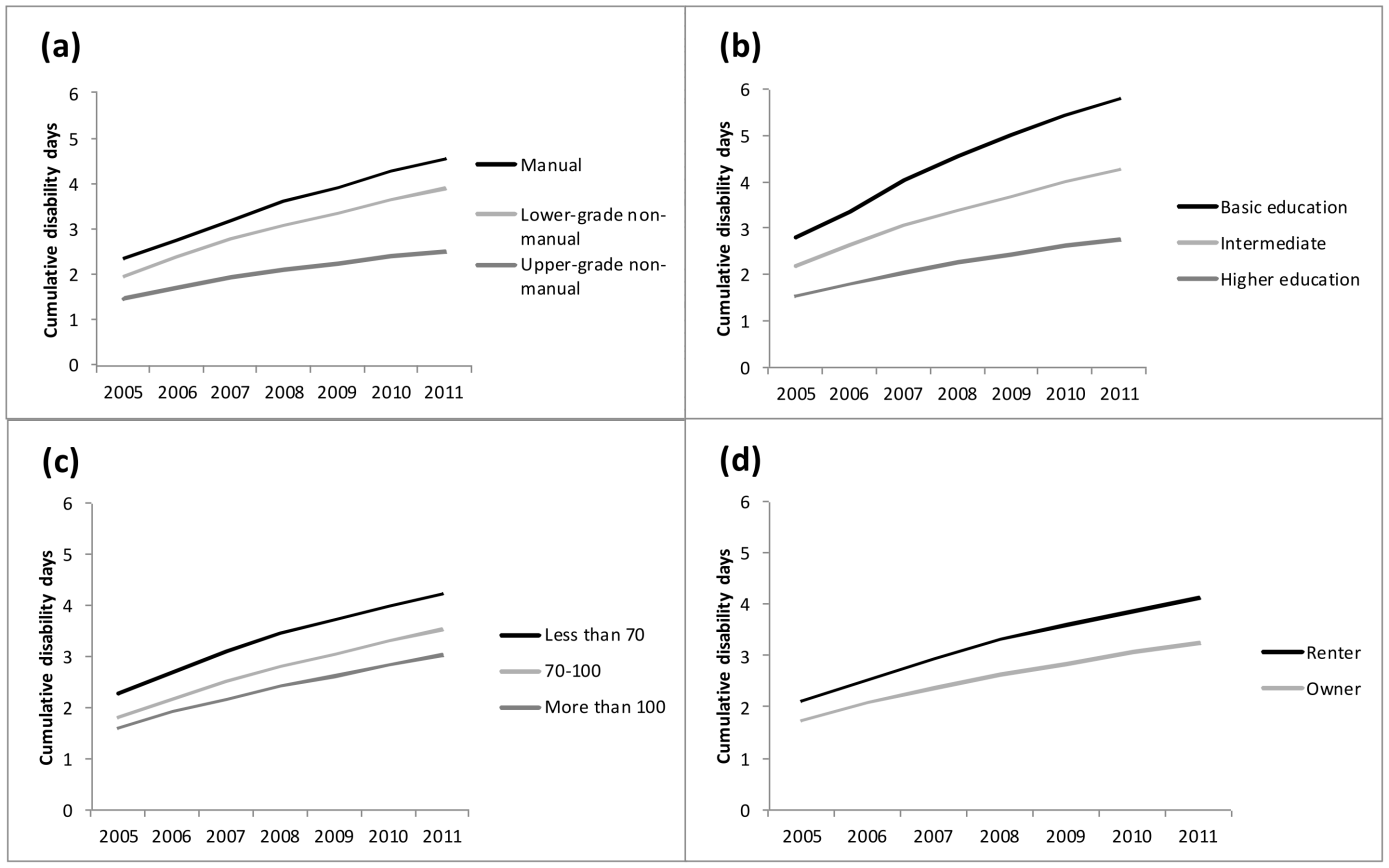
Cumulative disability days due to depression per cumulative person-years by indicators of socioeconomic status: (a) occupational position (b) level of education (c) residence size, m^2^ (d) residence ownership.

We found that work disability days due to depression accumulated more strongly in employees with lower SES. As illustrated in Figure 1, when examining cumulative disability days due to depression per cumulative person-years, the accumulation of depression-related work disability was more pronounced among employees in manual occupations compared to those in upper-grade non-manual occupations, and among employees with basic education compared to those with higher education. Moreover, employees in smaller and rented residences had more disability days per person-years than employees in larger and owner-occupied residences.

We found a socioeconomic gradient in work disability due to depression, irrespective of SES indicator (Table 2). Among upper-grade non-manual workers, mean of depression-related disability days per person-years was 2.5, whereas among lower-grade non-manual workers, it was 3.9 days, and among manual workers 4.6 days. When the SES indicators were entered separately into models adjusted for age and sex, manual workers were 1.96 times and lower-grade non-manual workers were 1.7 times more likely to suffer from work disability due to depression than upper-grade non-manual workers. Almost equally strong differences were found when using educational level, residence size, and residence ownership as a SES indicator.

**Table 2:** Associations between indicators of socioeconomic status (SES) work disability days due to depression in 2005–2011.

	Cum. disab.days^a^	Rate ratio (95% CI)	P-value	Rate ratio (95% CI)	P-value	Rate ratio (95% CI)	P-value
Indicator of SES		Separate models^b^		Model 1^c^		Model 2^d^	
Occupational position							
Upper-gradenon-manual	2.5	1 = Referent		1 = Referent		1 = Referent	
Lower-gradenon-manual	3.9	1.66 (1.47–1.86)	<0.001	1.40 (1.24–1.60)	<0.001	1.32 (1.17–1.50)	<0.001
Manual	4.6	1.96 (1.70–2.27)	<0.001	1.37 (1.15–1.64)	<0.001	1.35 (1.13–1.61)	<0.001
Education							
Higher	2.7	1 = Referent		1 = Referent		1 = Referent	
Intermediate	4.3	1.61 (1.44–1.79)	<0.001	1.35 (1.18–1.53)	<0.001	1.32 (1.17–1.50)	<0.001
Basic	5.8	1.89 (1.60–2.24)	<0.001	1.54 (1.27–1.88)	<0.001	1.52 (1.25–1.85)	<0.001
Residence size (m^2^)							
>100	3.0	1 = Referent		1 = Referent		1 = Referent	
70–100	3.5	1.26 (1.12–1.43)	<0.001	1.12 (0.99–1.27)	0.08	1.06 (0.94–1.20)	0.35
<70	4.2	1.70 (1.50–1.93)	<0.001	1.38 (1.20–1.58)	<0.001	1.23 (1.08–1.41)	0.002
Residence ownership							
Owner	3.2	1 = Referent		1 = Referent		1 = Referent	
Renter	4.1	1.53 (1.38–1.70)	<0.001	1.30 (1.16–1.45)	<0.001	1.30 (1.16–1.45)	<0.001

a Cumulative disability days due to depressive disorders per cumulative person-years in 2005–2011.

b Separate models (SES indicators entered separately) adjusted for age and sex.

c Model 1 adjusted for age and sex, and for all other SES indicators.

d Model 2 adjusted as model 1 and for presence of chronic somatic disease as well as work disability due to mental or behavioral disorder (ICD-10 codes F00–F99) in the previous year.

As also shown in Table 2, when all the SES indicators were simultaneously entered in the analysis the rate ratios for lower non-manual and manual occupations decreased to 1.4. Entering the presence of chronic somatic disease and prior mental disorder diagnosis into the model had little effect on these estimates. In the final model (Model 2) lower-grade non-manual workers were at a 1.3-fold, and manual workers were at a 1.4-fold risk of work disability due to depression when compared with upper-grade non-manual workers.

Although all SES indicators remained statistically significant predictors of disability days due to depression, the most powerful independent correlate was educational level. Compared to the highly educated, those with basic education only had a 1.5-fold, and those with intermediate education a 1.3-fold risk of work disability. A dose-response relationship was found with regard to educational level and residence size, while no such relationship was evident for occupational position.

### Socioeconomic Differences in RTW after Disability Episode due to Depressive Disorder

A total of 9 655 participants had at least one work disability episode of over nine days due to depression. Those disability episodes that could be followed-up for at least a year were included (13 701 episodes). Of these episodes, the majority (62%), ended in less than 2 months, 14% in 2–4 months, 8% in 4–8 months, 4% in 8–12 months, and 12% lasted longer than a year (these include episodes that did not end during the follow-up).

As shown in Table 3, when the SES indicators were entered separately into the model, manual work was associated with an increased risk of longer disability period, i.e., slower RTW compared to upper-grade non-manual work, COR = 1.2 (95% CI: 1.1–1.3); for basic education compared to higher education the corresponding COR was 1.3 (95% CI: 1.1–1.4). Residence size and ownership were not associated with time taken to RTW. In the final model adjusted for age, sex, other SES indicators, chronic somatic disease, and prior mental disorder diagnosis, the only significant predictor of RTW was educational level. Compared to those with higher education, those with basic education were at a 1.2-fold (95% CI: 1.1–1.4) risk of longer time until RTW.

**Table 3:** Associations between indicators of socioeconomic status (SES) and length of work disability due to depression.

	Cumulative odds ratio(95% CI)	P-value	Cumulative odds ratio(95% CI)	P-value	Cumulative odds ratio(95% CI)	P-value
Indicator of SES	Separate models^a^		Model 1^b^		Model 2^c^	
Occupational position						
Upper-grade non-manual	1 = Referent		1 = Referent		1 = Referent	
Lower-grade non-manual	0.97 (0.89–1.06)	0.51	0.96 (0.87–1.06)	0.39	0.94 (0.85–1.04)	0.24
Manual	1.17 (1.05–1.30)	0.006	1.10 (0.96–1.26)	0.16	1.09 (0.95–1.25)	0.20
Education						
Higher	1 = Referent		1 = Referent		1 = Referent	
Intermediate	1.03 (0.95–1.11)	0.51	1.01 (0.92–1.11)	0.85	1.01 (0.92–1.11)	0.84
Basic	1.27 (1.12–1.43)	<0.001	1.20 (1.05–1.38)	0.008	1.21 (1.05–1.39)	0.008
Residence size (m^2^)						
>100	1 = Referent		1 = Referent		1 = Referent	
70–100	0.92 (0.84–1.01)	0.09	0.91 (0.82–1.00)	0.04	0.90 (0.81–0.99)	0.03
<70	1.02 (0.93–1.12)	0.73	0.99 (89–1.09)	0.78	0.96 (0.87–1.07)	0.46
Residence ownership						
Owner	1 = Referent		1 = Referent		1 = Referent	
Renter	1.03 (0.96–1.11)	0.44	1.01 (0.93–1.09)	0.83	1.01 (0.93–1.09)	0.88

a Separate models (SES indicators entered separately) adjusted for age, sex, and year (when the disability begun).

b Model 1 adjusted for age, sex, and for all other SES indicators.

c Model 2 adjusted as Model 1 and for presence of chronic somatic disease as well as work disability due to mental or behavioral disorder (ICD-10 codes F00–F99) in the previous year.

### Socioeconomic Differences in Recurrence of Work Disability due to Depression

Forty five per cent (n = 6 423) of the participants who had returned to work from a previous disability period had recurrent episode of work disability due to depression. The time for the onset of a recurrent episode was 26 months on average (SD = 23). We found a significant, albeit small, socioeconomic gradient in the recurrence of work disability. When the SES indicators were entered separately into the model adjusted for age and sex, lower-grade non-manual (HR = 1.2, 95% CI: 1.1–1.3) and manual (HR = 1.2, 95% CI: 1.1–1.3) workers were at a higher risk of recurrent work disability than those in upper-grade non-manual positions (table 4). The risk of a recurrent episode was also higher for individuals with intermediate (HR = 1.1, 95% CI: 1.1–1.2) or basic (HR = 1.1, 95% CI: 1.0–1.2) education than for those with higher education, for individuals living in residences less than 70 m^2^ (HR = 1.1, 95% CI: 1.1–1.2) than for those living in residences larger than 100 m^2^, and for those living in rented residences (HR = 1.1, 95% CI: 1.0–1.1) than residence-owners.

**Table 4:** Associations between indicators of socioeconomic status and recurrence of work disability due to depression.

	N of events (%)	Hazard ratio(95% CI)	P-value	Hazard ratio(95% CI)	P-value	Hazard ratio(95% CI)	P-value
Indicator of SES		Separate models^a^		Model 1^b^		Model 2^c^	
Occupational position							
Upper-grade non-manual	1358 (41)	1 = Referent		1 = Referent		1 = Referent	
Lower-grade non-manual	3814 (47)	1.21 (1.13–1.30)	<0.001	1.17 (1.09–1.27)	<0.001	1.16 (1.07–1.25)	<0.001
Manual	1251 (46)	1.19 (1.09–1.30)	<0.001	1.13 (1.02–1.26)	0.02	1.14 (1.02–1.26)	0.02
Educational level							
Higher	2961 (44)	1 = Referent		1 = Referent		1 = Referent	
Intermediate	2767 (47)	1.11 (1.05–1.18)	<0.001	1.04 (0.97–1.11)	0.27	1.03 (0.96–1.10)	0.39
Basic	695 (47)	1.10 (1.00–1.21)	0.04	1.05 (0.94–1.17)	0.43	1.04 (0.93–1.15)	0.53
Residence size (m^2^)							
>100	1602 (44)	1 = Referent		1 = Referent		1 = Referent	
70–100	2382 (45)	1.05 (0.98–1.13)	0.20	1.02 (0.95–1.10)	0.59	1.01 (0.94–1.09)	0.89
<70	2439 (47)	1.13 (1.05–1.21)	0.001	1.09 (1.01–1.18)	0.02	1.06 (0.98–1.15)	0.13
Residence ownership							
Owner	3369 (44)	1 = Referent		1 = Referent		1 = Referent	
Renter	3054 (46)	1.07 (1.01–1.13)	0.02	1.03 (0.97–1.09)	0.40	1.02 (0.96–1.08)	0.51

a Separate models (SES indicators entered separately) adjusted for age and sex.

b Model 1 adjusted for age, sex, and for all other SES indicators.

c Model 2 adjusted as Model 1 and for presence of chronic somatic disease as well as work disability due to mental or behavioral disorder (ICD-10 codes F00–F99) in the previous year.

When all the SES indicators were entered simultaneously, and the model was also adjusted for all the other covariates, the only significant SES indicator predicting recurrent episodes was occupational position: participants in upper-grade non-manual positions were at a lower risk of a new disability episode than those in lower-grade non-manual (HR = 1.2, 95% CI: 1.1–1.3) and manual (HR = 1.1, 95% CI: 1.0–1.3) positions. The time for the onset of a recurrent episode was 28 months (SD = 24) for upper-grade non-manual workers, and 25 months (SD = 22) for lower-grade non-manual and manual workers. Residence size, residence ownership, or educational level, were not associated with recurrence of work disability due to depression (Table 4).

## Discussion

This prospective study of over 125 000 Finnish public sector employees examined the socioeconomic inequalities in work disability due to depression and found a consistent, albeit modest socioeconomic gradient in disability days, RTW after disability, and the recurrence of work disability due to depression. While Finland is a high-income European country with relatively equal welfare policy, it nevertheless has a marked socioeconomic gradient in health [24].

### Comparison with Previous Studies

Of the different SES indicators, educational level and occupational position were the strongest independent predictors of the disability outcomes used in this study. This finding was in contrast with a population-based cross-sectional Finnish study [7], which found income to be the most powerful correlate of a prevalent depressive disorder. Other studies have also found income to be a strong predictor of a prevalent depressive or anxiety disorder [8] and mortality [5]. However, our measure was different from these previous studies as we examined work disability as an outcome. It is also possible that our measure of accommodation as a proxy for income and wealth did not entirely capture the elements of social and material advantage or disadvantage.

Compared to the study by Virtanen et al. [9], which examined socioeconomic differences in very long-term (>90 days) work disability due to depression, we found slightly stronger associations suggesting larger socioeconomic differences in short-term compared to long-term work disability. However, with regard to socioeconomic differences in RTW, our estimates were lower than those reported by Virtanen et al. [9]. It is also possible that in less severe cases of depression, the socioeconomic differences in RTW after depression are smaller than in more severe cases.

Our findings are not in agreement with reports from the Netherlands and Denmark. In those studies, no association between SES and RTW was observed [12], [13] and, if anything, higher SES was associated with slower rather than faster RTW [10], [11]. The reason for these conflicting results is unknown but it is possible that differences in SES measures and outcome definition and chance or imprecision due to low statistical power in some studies have contributed to mixed evidence. It is also possible that differences in health care and disability benefit systems explain the observed variation in different studies. Thus, future studies comparing data from several countries would be of importance.

### Underlying Mechanisms and Implications

One explanation for the socioeconomic inequalities in depression-related work disability is the ‘Inverse care hypothesis’ [25] which suggests that there is a mismatch between the distribution of care and clinical need, and that treatment rates tend to be lower in the disadvantaged groups despite their higher disease rates and greater needs. Earlier studies have found that lower SES is linked with poorer care while higher SES and education have been associated with, for example, greater odds of visiting a psychotherapist [26], [27] and of taking antidepressant medication [28] when depressed. A recent study among UK participants [29] demonstrated a favorable trend in publicly provided psychotherapy and SES indicating that the treatment needs of those with the highest risk were better met in the public sector than previously. However, in that study privately provided psychotherapy treatment was still most common among high SES individuals, with no change over time.

Several other factors contributing to the socioeconomic differences in depression are also plausible. For example, comorbid physical and mental disorders are more common among individuals with lower SES; high SES individuals are more likely to have access to material resources they can use to access various forms of treatment (i.e., private sector treatment); social support, which is an important factor in the RTW and recovery from depression, may be poorer for low SES individuals; low SES individuals may have less control over their work schedules and arrangements to accommodate their health condition; and success of treatment may be lower for low SES individuals due to poorer treatment compliance and greater treatment resistance [9].

Implications for interventions based on observational data, such as ours, need to be considered with caution. A recent randomized controlled trial suggested that modification of job (compared with no modification) may facilitate reduction in symptoms among employees returning to work after depression [30]. Thus, modifiable work-related psychosocial factors are potentially important target for intervention. For example, job strain [31] characterized by a combination of high work demands, low job control, and low social support, has been linked with increased risk of depressive symptoms [32], [33] and excess risk of disability pension [34]. Moreover, employees with common mental disorders were more likely to feel unable to meet the demands of their job (RTW self-efficacy) if they had fast work pace and heavy workload before sick leave [35]. As job strain and other work-related risk factors tend to be more common among employees with low SES, they provide a potentially relevant target for interventions to reduce the socioeconomic gradient in depression. Indeed, some workplace interventions have shown reductions in depressive symptoms among employees although the effects on RTW have been modest [36].

### Strengths and Limitations

The study benefits from its large sample size, prospective study design, and reliable register data, which is assumed to be free from many biases related to self-report data. We contributed to existing evidence on the socioeconomic gradient in depression-related work disability and its prognosis by examining, for the first time, multiple SES indicators, and the multiple outcomes of work disability due to depression.

The study also has some limitations. First, although we had data on several SES indicators, we had no direct data on participants’ personal or family income or wealth. However, we used information on accommodation (residence size and residence ownership) as a proxy for income and wealth. Second, despite the prospective design, we were unable to control for all previous episodes of work disability due to depression during the life course, or early selection of lower education and lower-grade jobs owing to depression or other mental disorders. Moreover, although many covariates were adjusted for in the statistical models, we did not have data on all potential confounders, such as depression severity, marital status, and psychosocial work conditions, which have been linked to RTW and the recurrence of depression in previous studies [12]. Third, our study population was comprised of Finnish public sector employees which may limit the generalizability of our findings. Further research is needed to examine whether similar findings are obtained from general population samples and other countries with different benefit systems. Future research should also examine the mechanisms through which the socioeconomic differences impact depression, RTW, and recurrence.

## Conclusions

This study of Finnish public sector employees suggests socioeconomic inequalities in the onset and recovery from work disability due to depression. Our findings suggest that socioeconomic status, especially occupational position and educational level, should be taken into account in the attempts to reduce social inequalities in work disability due to depression. However, further research is needed to examine the generalizability of our finding across different populations.
